# Effects of elevated root-zone CO_2_ on nutrient uptake and rhizosphere microbiome in Oriental Melon (Cucumis melo var. makuwa Makino)

**DOI:** 10.3389/fpls.2026.1888248

**Published:** 2026-08-31

**Authors:** Xiaoxue He, Feng Li, Xintong Han, Ying Liu, Shuai Pei, Ziqi Wang, Zhiyong Xie, Yiling Liu

**Affiliations:** 1 College of Horticulture, Shenyang Agricultural University/Key Laboratory of Protected Horticulture, Ministry of Education/National & Local Joint Engineering Research Center of Northern Horticultural Facilities Design & Application Technology, Shenyang, Liaoning, China; 2 Chengde Academy of Agriculture and Forestry Sciences (Chengde AAFS), Chengde, Hebei, China; 3 Suizhong County Agricultural Affairs Service Center, Huludao, Liaoning, China; 4 Shenyang Institute of Technology (SIT), Shenyang, Liaoning, China

**Keywords:** elevated root-zone CO_2_, growth performance, nutrient uptake, Oriental melon, rhizosphere microbial dynamics

## Abstract

Despite the significance of CO_2_ as a crucial gaseous component in the soil environment, the effects of increased root-zone CO_2_ concentrations on the physiological responses of melon and the corresponding rhizosphere microbiome are still not well understood. This study examined the effects of elevated root-zone CO_2_ on oriental melon (Cucumis melo var. makuwa Makino) (‘Yu Mei Ren’) in a pot experiment under controlled O_2_ conditions. Treatments included a control group (3000 μmol·mol^-^¹ CO_2_) and three elevated concentrations: T1 (6000 μmol·mol^-^¹), T2 (9000 μmol·mol^-^¹), T3 (12000 μmol·mol^-^¹). This study assessed plant growth, nutrient uptake, and rhizosphere soil properties, encompassing physicochemical characteristics and microbial community structure. The results indicated that at 10 days of treatment, T1, T2, and T3 significantly suppressed melon growth and nutrient assimilation. Additionally, a reduction in rhizosphere soil pH and an increase in electrical conductivity (EC) were noted. These differences became increasingly pronounced with extended treatment. Analysis of the rhizosphere microbial community indicated that elevated root-zone CO_2_ decreased both diversity and richness, while simultaneously increasing the relative abundance of the phyla Acidobacteria and Actinobacteria, along with genera such as SBR1031 and A4b. These results indicate that long-term elevated root-zone CO_2_ not only induces soil acidification and alters microbial community structure, but also impairs root nutrient acquisition.

## 1 Introduction

A well-maintained root zone is essential for plant growth and development, playing a critical role throughout the life cycle (Chen et al., 2020; Teskey et al., 2008; Shimono et al., 2019; Byun et al., 2020). Among the various factors affecting the root zone, dynamic changes in rhizospheric gaseous components—such as O_2_, CO_2_, and N_2_O—significantly influence plant growth and nutrient acquisition. The atmospheric concentraton of CO_2_ near the Earth’s surface usually hovers around 400 μmol·mol^-^¹. However, soil CO_2_ levels frequently exceed this concentration due to the processes of root and microbial respiration. This increased CO_2_ in the root zone serves as a vital carbon source for soil microorganisms (Jin et al., 2022). Rhizosphere microorganisms are essential for sustaining biological nitrogen fixation, facilitating nutrient cycling, and regulating greenhouse gas emissions, forming a dynamic interaction network with plants (Siqinbatu et al., 2013; Mueller et al., 2018). Furthermore, plants release sugars and organic acids via root exudates, which serve as carbon sources for microbes, attract specific plant growth-promoting bacteria, and stimulate root meristem activity, thereby enhancing lateral root density (Tfaily et al., 2018). In poorly aerated soils, localized high concentrations of CO_2_ can negatively impact the stability of the rhizosphere microbial community composition (Dan et al., 2014). In rice, low O_2_ stress results in reduced root biomass, morphological alterations, and decreased nutrient absorption and translocation, accompanied by a significant increase in anaerobic bacteria (Jian et al., 2025; Williams et al., 2000). This stressor compels plants to enter an energy intensive defense mode, which directly suppresses growth, while the concurrently altered rhizosphere environment destabilizes the microbial community, further jeopardizing plant health (Kai et al., 2024). Although CO_2_ is essential for plant metabolism and a key respiratory product of soil microbes, excessive CO_2_ accumulation in the root zone may act as a major environmental stressor, adversely affecting plant performance. Recently, Chen et al. (2020) and Han et al. (2022) reported the effects of elevated root-zone CO_2_ on root morphology and carbon partitioning in melon seedlings, but their studies did not address the concomitant changes in nutrient uptake or the compositional shifts of the rhizosphere microbial community. Given that the rhizosphere microbiome plays a critical role in nutrient cycling and plant health (Hinsinger et al., 2009; Mueller et al., 2018), the specific mechanisms by which elevated root-zone CO_2_ affects nutrient acquisition and rhizosphere microbial communities in melon remain poorly understood.

This study examined the effects of four distinct root-zone CO_2_ concentrations on the growth, rhizosphere soil properties, and microbial community composition of the oriental melon ‘Yu Mei Ren’. This cultivar is widely grown in solar greenhouses in northern China, where poor soil aeration under protected cultivation frequently leads to CO_2_ accumulation. The objective was to clarify the influence of elevated soil CO_2_ on nutrient uptake and the root-zone microbiome, thereby offering a theoretical foundation for enhancing protected melon cultivation in conditions of inadequate soil ventilation. These findings may provide practical guidance for mitigating rhizosphere CO_2_-related stress in protected melon cultivation.

## 2 Materials and methods

### 2.1 Experimental materials

Seedlings were raised in a solar greenhouse under natural light conditions. During the seedling stage, the daytime temperature ranged from 25 °C to 30 °C, and the nighttime temperature ranged from 18 °C to 24 °C. Seedlings were irrigated with tap water as needed, and no additional fertilizer was applied before transplanting.

The experiment was conducted in a solar greenhouse at Shenyang Agricultural University’s research base. Oriental melon (*Cucumis melo* var. *makuwa* Makino) *Cucumis melo* ‘Yu Mei Ren’ was used as plant material, with seeds provided by Hengyuan Seeds Co., Ltd. (Changchun, China). During the growth period, the daytime temperature ranged from 25 to 30 °C, and the nighttime temperature was controlled between 18 and 24 °C. The relative humidity in the greenhouse was maintained within the range of 60%–85% throughout the cultivation period. Regarding light conditions, natural light was used as the light source without supplemental artificial lighting (i.e., natural daylight). The experiment was conducted under ambient light conditions characteristic of the growing season and geographical location.

### 2.2 Experimental methods

**Figure 1:**
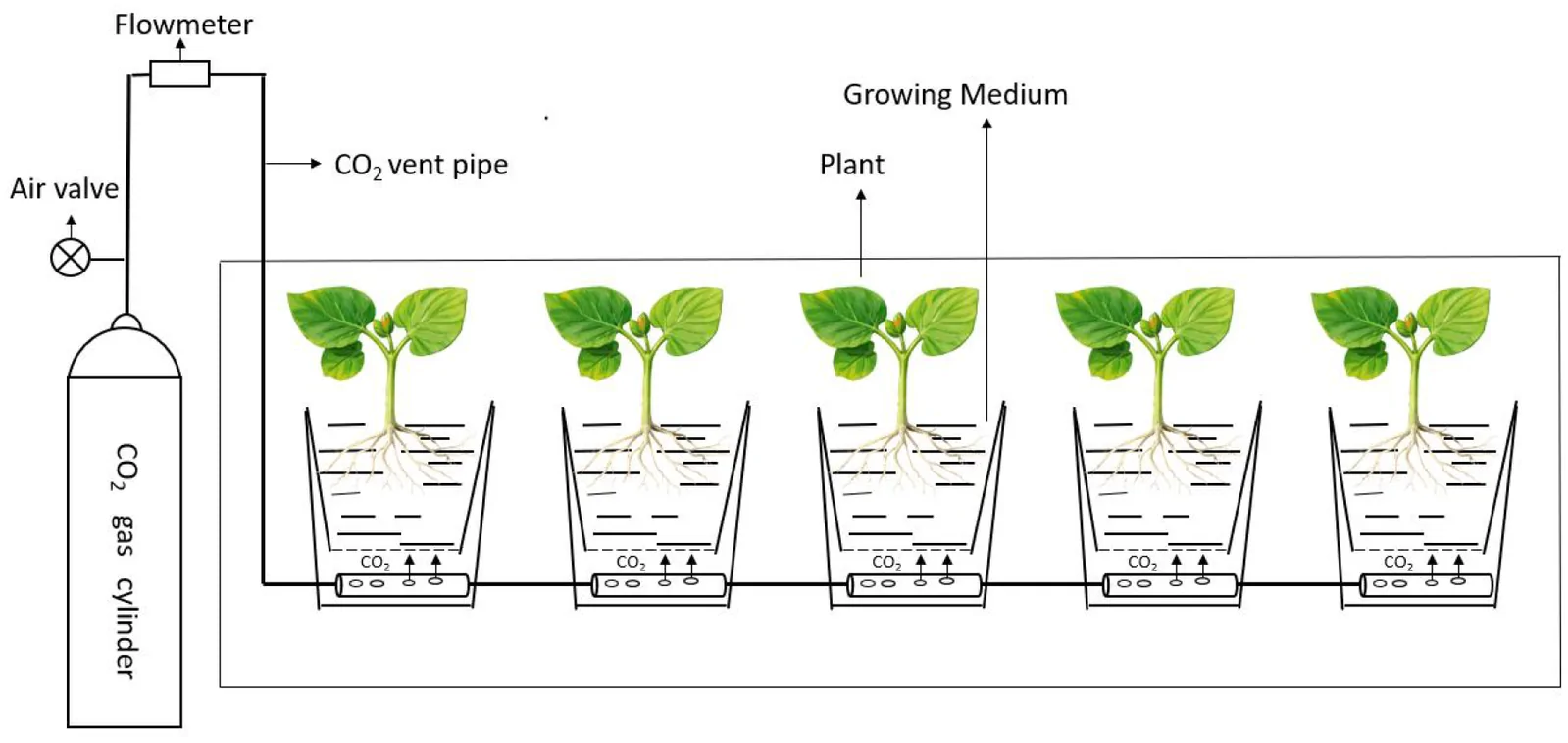
Diagram of CO_2_ treatments in the root zone. The system consisted of a CO_2_ gas cylinder, a gas flow meter, a CO_2_ sensor, and automated control valves to regulate CO_2_ concentration in the root zone. The cultivation trough (200 cm × 30 cm × 30 cm) was filled with growth substrate. Seedlings of oriental melon (Cucumis melo var. makuwa Makino) (‘Yu Mei Ren’) were planted at a spacing of 20 cm.

At the three-true-leaf stage, selected uniform and healthy seedlings were transplanted into cultivation troughs (200 cm L × 30 cm W × 30 cm H; **Figure 1**). A specific quantity of substrate was added to each trough. The plants were arranged with a spacing of 20 cm, resulting in a total of 10 plants per trough, and the entire setup was replicated three times. CO_2_ treatments began 7 days after transplanting, at the four-true-leaf stage, utilizing a controlled-release system adapted from Zhang (2017). This system, which included gas tubing, a flow meter, a CO_2_ cylinder, and a CO_2_ sensor, automatically regulated the concentration through a solenoid valve (**Figure 1**). The main components of the growing substrate are cow manure and peat, and its physical and chemical properties are shown in **Table 1**.

**Table 1:** Nutrient content of test substrate.

pH value	EC value	Total N		Total	Total	Available N	Available P	Available K
	(dS/m)		(g/kg)	P(g/kg)	K(g/kg)	(mg/kg)	(mg/kg)	(mg/kg)
7.48	2.15		11.67	3.16	23.73	180.57	176.42	1884.33

Based on preliminary group monitoring that established a normal root-zone CO_2_ range of 3000–6000 μmol·mol^-^¹ in oriental melon seedlings, 3000 μmol·mol^-^¹ was designated as the control level. Three elevated treatments were then implemented: T1 (6000 μmol·mol^-^¹), T2 (9000 μmol·mol^-^¹), and T3 (12,000 μmol·mol^-^¹), all maintained under a constant O_2_ level of 21%. O_2_ concentration was monitored using a BH-4C portable handheld detector, with a single-point measurement taken before each CO_2_ treatment (non-continuous monitoring). To overcome the decrease in O_2_ concentration caused by root respiration during the experiment, air (containing 21% O_2_) was supplied at an equal flow rate simultaneously with the introduction of CO_2_. Through this dynamic compensation of the gas mixture, the rhizosphere O_2_ concentration was maintained at a constant level.

### 2.3 Measurement indicators and methods

#### 2.3.1 Sample collection

Plant samples were collected at 0, 10, 20, and 30 days post-treatment by randomly selecting three uniformly grown individuals at each time point. The harvested plants were rinsed with distilled water, dissected into roots, stems, and leaves, and subsequently heat-inactivated at 105 °C for 15 minutes. Drying was performed in an oven at 80 °C until a constant weight was attained, followed by grinding, sieving through a 100-mesh sieve, and storage in sealed containers.

Rhizosphere soil (0–20 cm depth) was collected at the conclusion of the 30-day treatment. For microbial community analysis, a 10 g subsample was immediately flash-frozen in liquid nitrogen, stored at -80 °C, and subsequently utilized for DNA extraction and 16S rRNA gene sequencing.

#### 2.3.2 Determination of growth parameters and nutrient content in oriental melon plants

Plant height was measured from the stem base to the apical meristem, while stem diameter was assessed using a digital caliper. After harvest, fresh weight was recorded following the blotting of surface moisture. For dry weight determination, samples were initially oven-fixed at 105 °C for 30 minutes and subsequently dried at 65°C until reaching a constant weight. All weights were measured with a precision balance after the samples were cooled in a desiccator. Root activity was assessed using the 2,3,5-triphenyltetrazolium chloride (TTC) reduction metho (Xie et al., 2020), and photosynthetic pigment levels were determined following extraction in a 1:1 (v/v) acetone: ethanol solution (Ritchie, 2006). Photosynthetic parameters were measured on clear mornings between 9:00 and 11:30 a.m. using a Li-6800 portable photosynthesis system (Chen et al., 2022). Fully expanded functional leaves at the third node below the growing point were selected for the measurements. For each treatment, three randomly selected plants with uniform growth served as biological replicates, and all parameters were measured in triplicate for each replicate.

Plant nutrient content was assessed according to the methodologies outlined by Zou (2000) and Bao (2005). Samples were dried, ground, and digested using the H_2_SO_4_-H_2_O_2_ procedure (Thomas et al., 1967). After digestion, total nitrogen was quantified using a Kjeldahl analyzer (Kjeldahl, 1883), total phosphorus was determined through vanadate-molybdate colorimetry (Kitson and Mellon, 1944), and total potassium was measured by flame photometry (Issac and Johnson, 1976).

#### 2.3.3 Determination of substrate physicochemical properties

Soil pH and EC were measured in a 1:5 (w/v) soil-water suspension after shaking for 30 min and filtration, using a pH/EC meter. Soil organic matter was quantified using the K_2_Cr_2_O_7_ volumetric method. The determination of alkali-hydrolyzable nitrogen, available phosphorus, and available potassium were conducted following the procedures outlined by Bao (2000), employing alkali diffusion, molybdenum-blue colorimetry, and flame photometry methods, respectively.

#### 2.3.4 Determination of rhizosphere microbiota

For rhizosphere soil sampling, loosely attached bulk soil was first removed from the roots by gentle shaking, and the soil tightly adhering to the root surface was then collected using a sterile soft-bristle brush and passed through a 100-mesh sieve (150-μm pore size). One subsample (10 g) was immediately flash-frozen in liquid nitrogen and stored at −80 °C for subsequent DNA extraction and microbial sequencing analysis. Another subsample was air-dried at room temperature, passed through a 1-mm sieve, and stored at room temperature for physicochemical property determination. Amplicon sequencing of the 16S rRNA gene was performed on the Illumina MiSeq platform by Shanghai Majorbio Bio-Pharm Technology Co., Ltd (Shanghai, China).

For enumeration of culturable microorganisms, 10 g of fresh rhizosphere soil was suspended in 90 mL sterile water and serially diluted (10^-^¹ to 10^-5^). Aliquots (0.1 mL) were spread on selective media: beef extract-peptone agar for bacteria (incubated at 37 °C for 2 days), Martin’s agar for fungi (28 °C for 5 days), and Gause’s No. 1 agar with 3% K_2_Cr_2_O_7_ for actinomycetes (28 °C for 7 days). Colonies were counted on plates with 30–300 CFU. Results were expressed as CFU per gram of dry soil. Three biological replicates were used (Yuan et al., 2025).

#### 2.3.5 DNA extraction

Total DNA was extracted from substrate samples utilizing the Fast DNA Spin Kit for Soil (MP Biomedicals). The quality of the extracted DNA was evaluated through electrophoresis on a 1% agarose gel.

#### 2.3.6 PCR amplification and purification

Prior to sequencing, three DNA aliquots from each sample were homogenized and pooled to serve as the PCR template. The V3-V4 hypervariable region of the bacterial 16S rRNA gene was amplified using universal primers 338F (5′-ACTCCTACGGGAGGCAGCAG-3′) and 806R (5′-GGACTACHVGGGTWTCTAAT-3′). The amplified products were purified using the Agarose Gel DNA Purification Kit (Takara). Purified amplicons were evaluated with a NanoDrop 2000 spectrophotometer and analyzed by 2% agarose gel electrophoresis. Qualified products were utilized to construct paired-end (PE) libraries for sequencing on the Illumina MiSeq platform. The composition of the PCR reaction mixture is detailed in **Tables 2**, **3**.

**Table 2:** PCR reaction system.

Reagent(s)	Dosage
5×FastPfu Buffer	4μL
2.5mM dNTPs	2μL
Forward Primer(5μM)	0.8μL
Reverse Primer(5μM)	0.8μL
Fast Pfu Polymerase	0.4μL
BSA	0.2μL
Template DNA	10ng
ddH_2_O	Bring up to 20μL

**Table 3:** PCR reaction parameters.

Procedure	Duration	Temperature	Cycles
Initial Denaturation	3min	95°C	1
Denaturation	30 s	95°C	35
Annealing	45 s	60°C	35
Extension	120 s	72°C	35
Storage Conditions	∞	10°C	—

Taxonomic analysis:(1) Low-quality sequences were filtered; (2) High-quality sequences were clustered into operational taxonomic units (OTUs) at a 97% similarity threshold; (3) Chimeras were removed, and sequences were rarefied to the minimum sample depth. OTU representative sequences (97% similarity) were taxonomically classified using the RDP classifier Bayesian algorithm. Community composition was statistically analyzed at each taxonomic level with reference to the SILVA database (Release 138, http://www.arb-silva.de).

### 2.4 Statistical and bioinformatics analysis

All data are presented as mean ± standard deviation. Data processing was performed using Excel 2019, and statistical analysis was conducted using SPSS 26.0 software. First, normality tests and homogeneity of variance tests were performed on each dataset. If the data met the assumptions of normal distribution and homogeneity of variance, one-way ANOVA was used, pearson product-moment correlation coefficient was used for correlation analysis, and a two-tailed test was applied, with the significance level set at *p* < 0.05. Amplicons were purified and sequenced on an Illumina MiSeq platform (2 × 300 bp). Sequence data were processed using QIIME2. OTUs were clustered at 97% similarity. Taxonomy was assigned using the RDP classifier against the SILVA v138 database. Alpha diversity indices (Chao1, Shannon, Coverage) were calculated in QIIME2.

## 3 Results

### 3.1 Elevated root-zone CO_2_ concentrations significantly inhibited the growth performance of oriental melon

**Figure 2:**
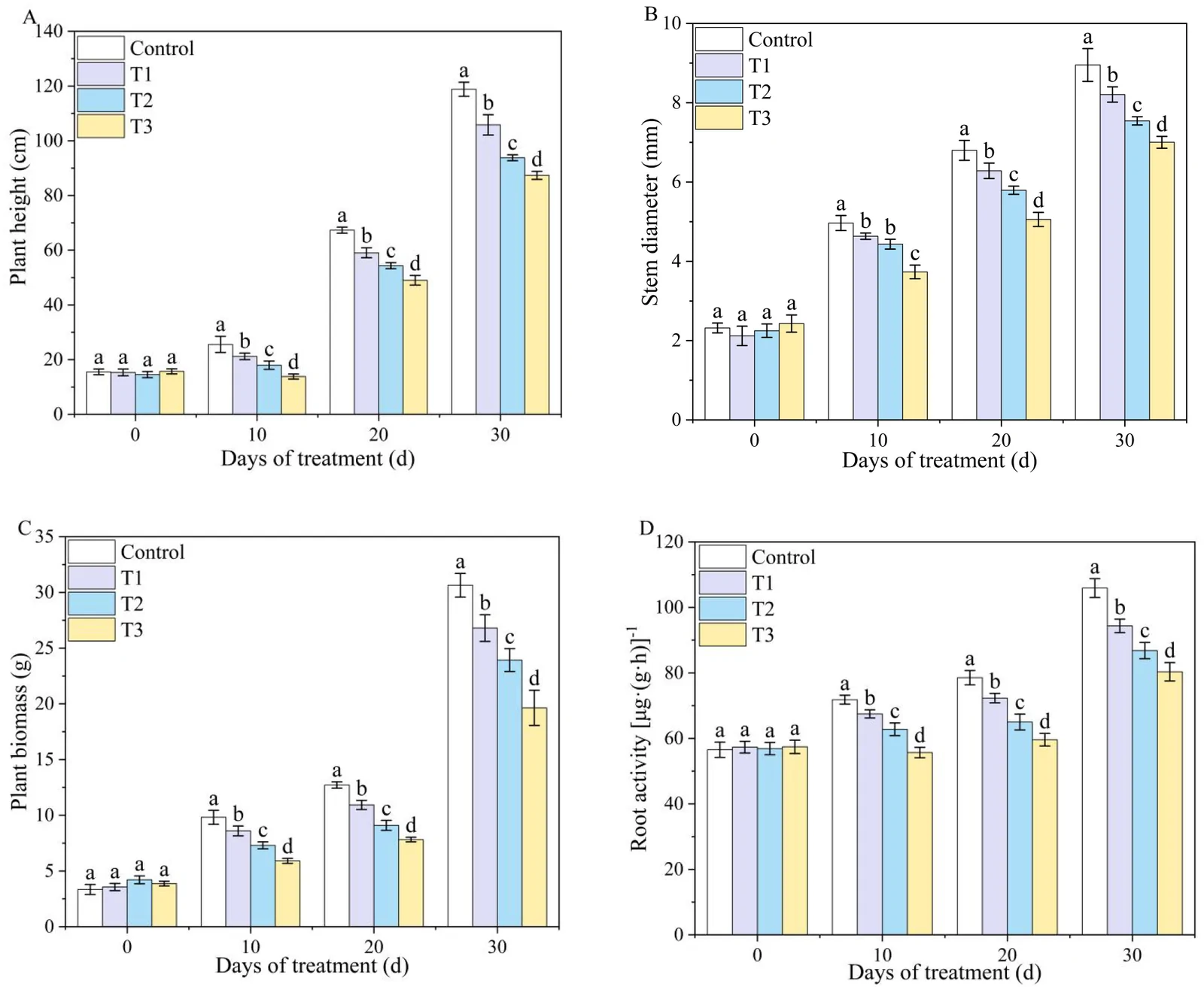
Effect of high soil environmental concentration of CO_2_ on the growth of oriental melon seedlings. **(A)** Plant height (cm); **(B)** Stem diameter (mm); **(C)** Total biomass (g·plant^-^¹, dry weight); **(D)** Root activity (μg·g^-^¹·h^-^¹, fresh weight). Treatments: Control (3000 μmol/L CO_2_), T1 (6000 μmol/L), T2 (9000 μmol/L), T3 (12000 μmol/L). Oxygen concentration was maintained at 21% for all treatments.

The growth status of oriental melon, evaluated through plant height, stem diameter, biomass, and root activity, demonstrated a clear concentration-dependent suppression under elevated root-zone CO_2_ on day 10 (**Figure 2**). All treatment groups (T1-T3) exhibited significant reductions in these metrics compared to the control, with the suppression becoming more pronounced at higher CO_2_ levels. The most substantial effects were noted in plant height and biomass, which decreased by 45.9% and 39.8%, respectively, under the T3 treatment. This indicates that growth and dry mass accumulation are particularly sensitive to elevated root-zone CO_2_ levels.

### 3.2 Elevated root-zone CO_2_ significantly reduced the photosynthetic parameters in the leaves of oriental melon

**Figure 3:**
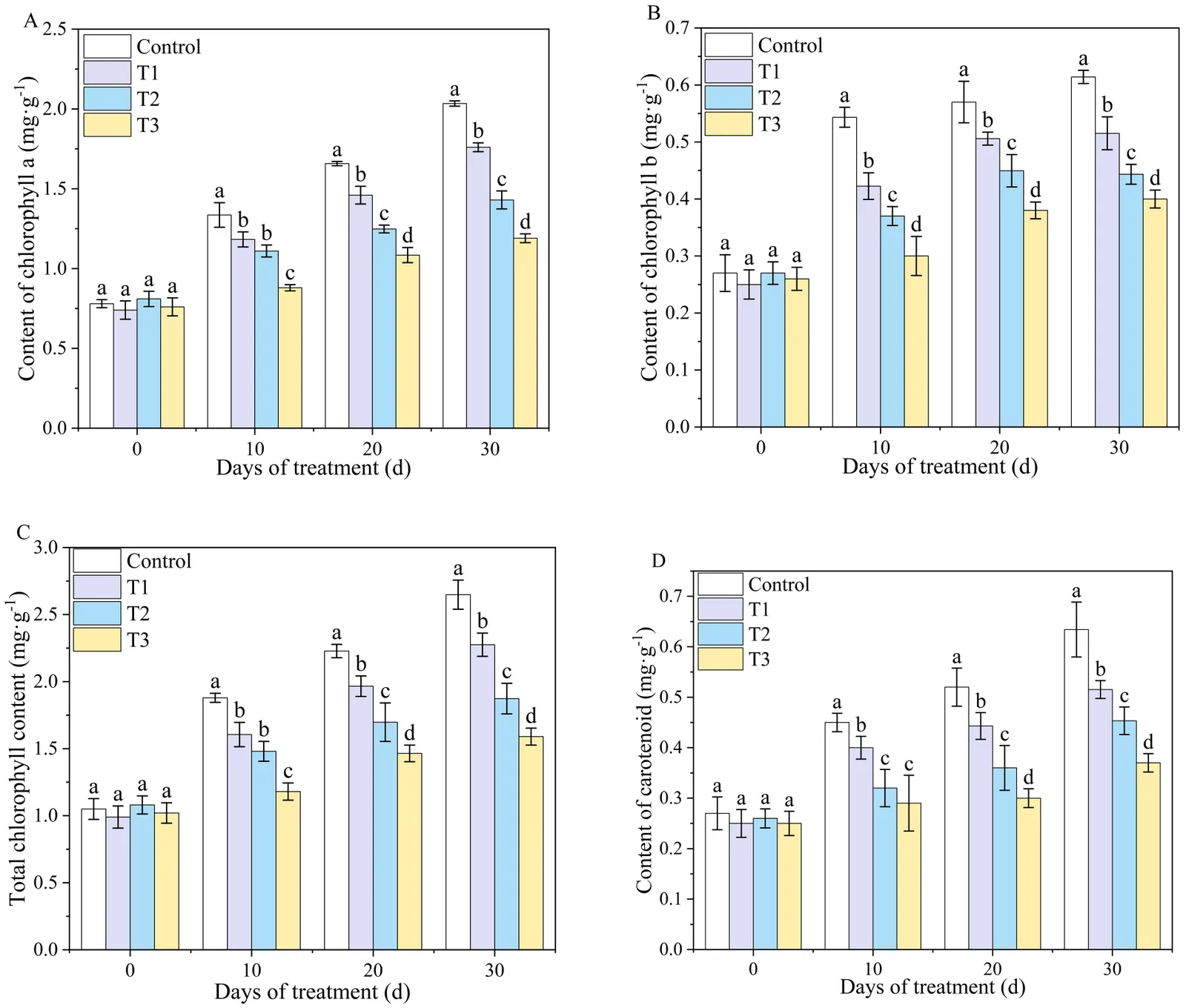
Effect of high concentration of CO_2_ in root zone soil environment on photosynthetic pigment in oriental melon seedlings. **(A)** Chlorophyll a content (mg·g^-^¹ FW); **(B)** Chlorophyll b content (mg·g^-^¹ FW); **(C)** Total chlorophyll content (mg·g^-^¹ FW); **(D)** Carotenoid content (mg·g^-^¹ FW). Treatments: as in **Figure 2**. Data presentation: Bars represent means ± SD (n=3). Different lowercase letters above bars indicate significant differences among treatments at P<0.05 (one-way ANOVA followed by Duncan’s multiple range test). FW, fresh weight; SD, standard deviation.

Photosynthetic pigments, essential for the conversion of light energy, demonstrated significant sensitivity to root-zone CO_2_ stress, with notable reductions evident by day 10 (**Figure 3**). Compared to the Control, all elevated CO_2_ treatments (T1-T3) resulted in significant decreases in the concentrations of chlorophyll a (Chl a, **Figure 3A**), chlorophyll b (Chl b, **Figure 3B**), total chlorophyll (Total Chl, **Figure 3C**), and carotenoid (Car, **Figure 3D**), revealing a strong negative correlation between pigment content and CO_2_ levels. The T3 treatment induced the most pronounced declines: 34.3% for Chl a, 44.4% for Chl b, 37.2% for total Chl, and 35.6% for Car. These findings suggest that elevated root-zone CO_2_ likely disrupts chlorophyll biosynthesis, posing a immediate threat to the integrity of the photosynthetic machinery.

**Figure 4:**
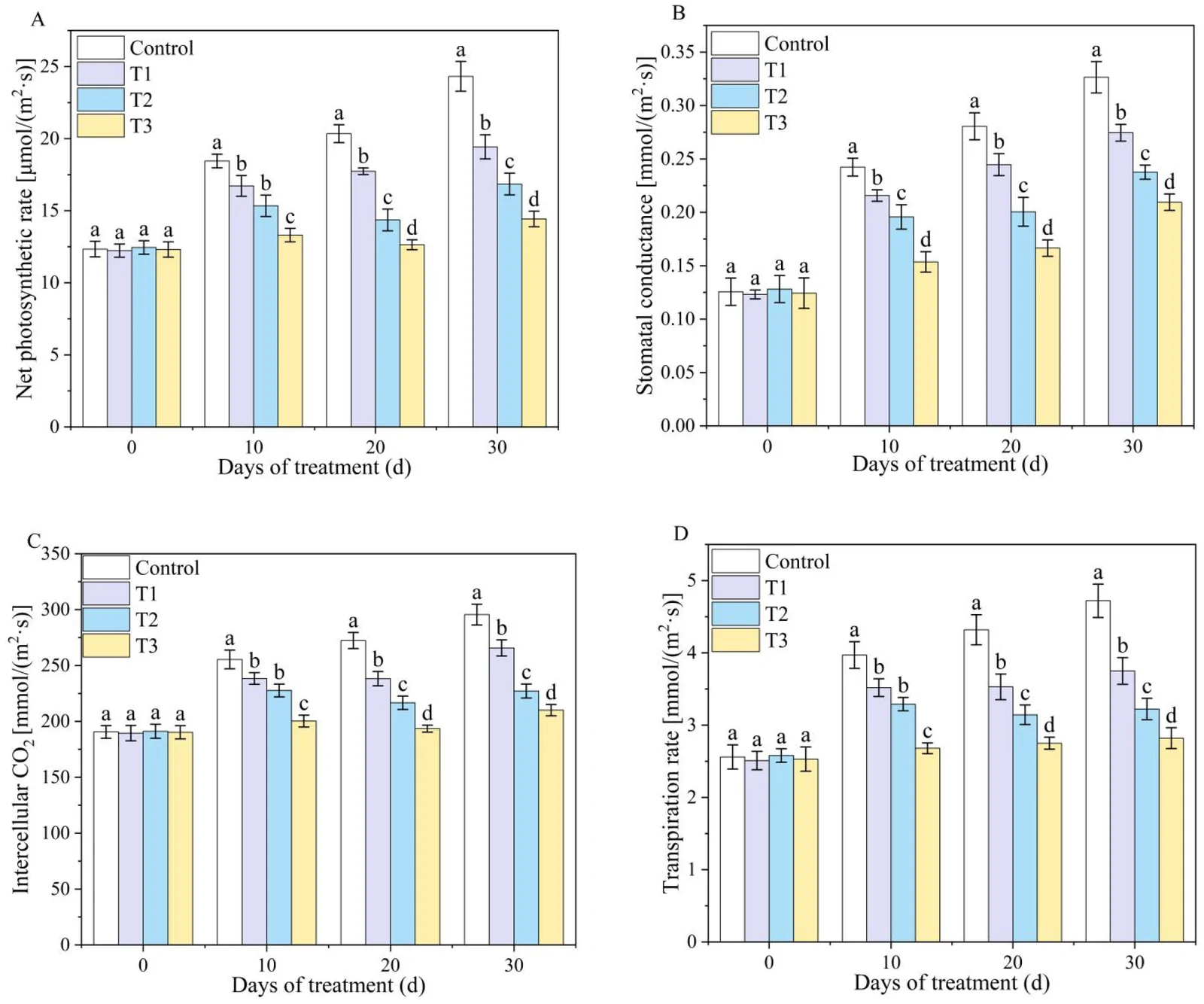
Effect of high concentration of CO_2_ in root zone soil environment on photosynthetic parameters in oriental melon seedlings. **(A)** Net photosynthetic rate (Pn, μmol CO_2_·m^-^²·s^-^¹); **(B)** Stomatal conductance (Gs, mol H_2_O·m^-^²·s^-^¹); **(C)** Intercellular CO_2_ concentration (Ci, μmol CO_2_·mol^-^¹); **(D)** Transpiration rate (Tr, mmol H_2_O·m^-^²·s^-^¹). Treatments: as in **Figure 2**. Oxygen concentration was maintained at 21% for all treatments. Data presentation: Bars represent means ± SD (n=3). Different lowercase letters above bars indicate significant differences among treatments at P<0.05 (one-way ANOVA followed by Duncan’s multiple range test). Pn, net photosynthetic rate; Gs, stomatal conductance; Ci, intercellular CO_2_ concentration; Tr, transpiration rate; SD, standard deviation.

Leaf photosynthetic parameters exhibited a significant decline under elevated root-zone CO_2_ at day 10 (**Figure 4**). The values for net photosynthetic rate (Pn), stomatal conductance (Gs), intercellular CO_2_ concentration (Ci), and transpiration rate (Tr) were significantly lower in treated plants compared to controls. The coordinated decrease in Gs and Ci suggests that stomatal limitation is a primary factor constraining photosynthesis. Although parameters did not differ significantly between T1 and T2, the T3 treatment resulted in the most pronounced inhibition, reducing Pn by 27.9% and thereby indicating a strong, concentration-dependent suppression of photosynthesis by root-zone CO_2_.

### 3.3 Elevated root-zone CO_2_ significantly reduced nutrient accumulation in oriental melon plants

**Figure 5:**
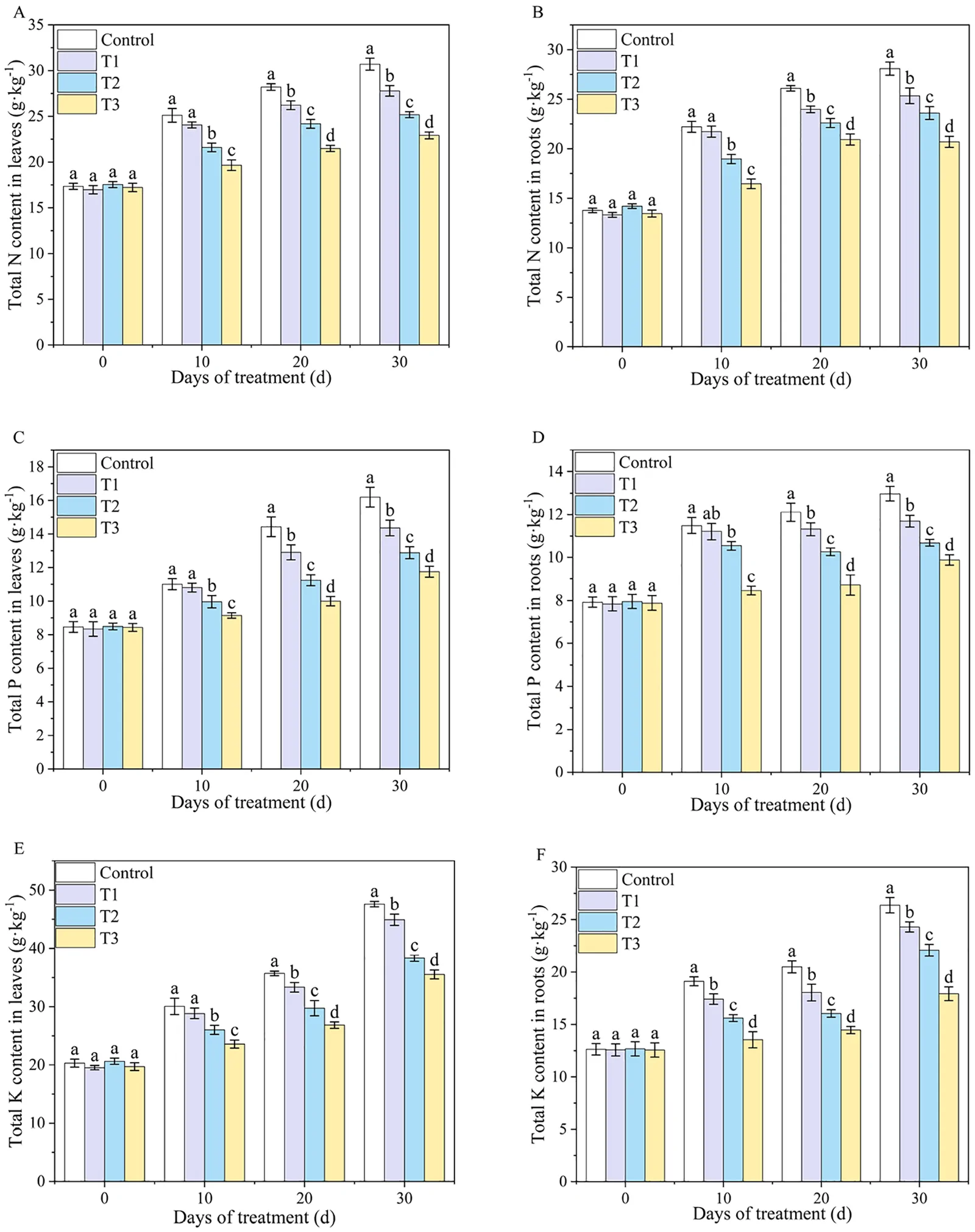
Effect of high soil concentration CO_2_ on accumulation of nutrient content in oriental melon seedlings. **(A)** Total nitrogen in leaves (g·kg^-^¹ DW); **(B)** Total nitrogen in roots (g·kg^-^¹ DW); **(C)** Total phosphorus in leaves (g·kg^-^¹ DW); **(D)** Total phosphorus in roots (g·kg^-^¹ DW); **(E)** Total potassium in leaves (g·kg^-^¹ DW); **(F)** Total potassium in roots (g·kg^-^¹ DW). Treatments: as in **Figure 2**. Data presentation: For each time point, bars = mean ± SD (n = 3). Different lowercase letters indicate significant differences among treatments at P<0.05 (ANOVA + Duncan). DW, dry weight.

Nutrient accumulation in oriental melon seedlings exhibited a dual dependency, decreasing with both rising CO_2_ concentration and prolonged treatment duration (**Figure 5**). At 10 days post- treatment, significant reductions in total nitrogen (N, **Figures 5A, B**), total phosphorus (P, **Figures 5C, D**), and total potassium (K, **Figures 5E, F**) in both leaves and roots were observed specifically in the T2 and T3 treatments. The inhibitory effect of T3 was more pronounced than that of T2, with T3 resulting in a maximum reduction of 32.0% in root N. The T1 treatment did not differ statistically from the Control. By day 20, a clear dose-dependent inhibition emerged across all treatments (T3>T2>T1), indicating that prolonged root-zone CO_2_ stress chronically disrupts nutrient acquisition and partitioning.

### 3.4 Elevated root-zone CO_2_ significantly altered the pH, electrical conductivity, and nutrient content of the rhizosphere soil in oriental melon

**Figure 6:**
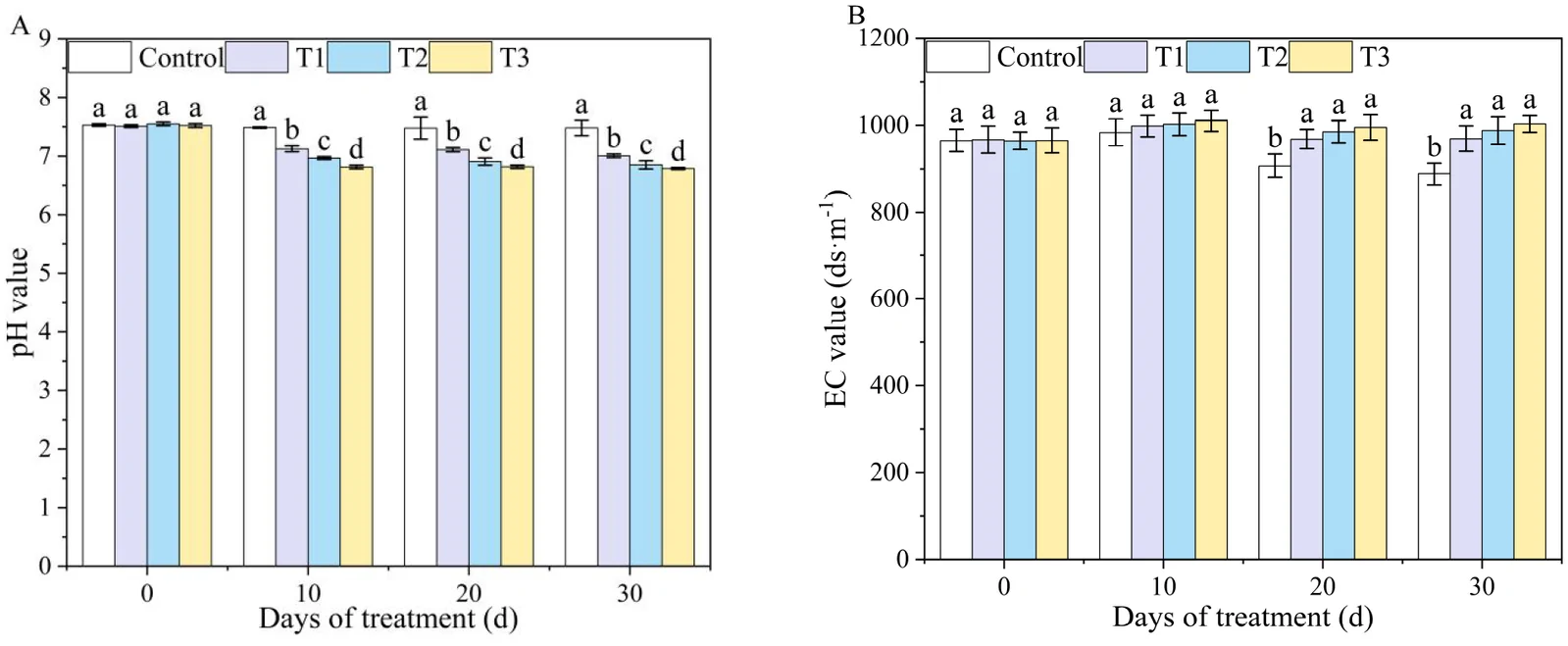
Effect of high soil environment CO_2_ on matrix pH and EC in the root region of oriental melon seedlings. **(A)** Soil pH; **(B)** Electrical conductivity (EC, ds·m^-^¹). Samples taken at 10, 20, and 30 days after treatment. Treatments: as in **Figure 2**. Data presentation: Bars = mean ± SD (n = 3). Different letters indicate significant differences at P<0.05 (ANOVA + Duncan). EC, electrical conductivity.

The properties of rhizosphere soil exhibited divergent temporal responses to elevated CO_2_ (**Figure 6**). At 10 days post-treatment, soil pH (**Figure 6A**) significantly decreased across all treatment groups (T1, T2, T3) compared to the Control, with reductions of 4.8%, 6.9%, and 9.1%, respectively. This finding indicates a rapid acidification effect induced by the high CO_2_ environment. Conversely, electrical conductivity (EC, **Figure 6B**) showed a delayed response, becoming significantly elevated only by day 20 across all treatments. This observation suggests that the accumulation of soluble ions, potentially resulting from microbial metabolic activity or root exudation, occurs gradually. These temporal dynamics reflect the progressive and persistent alterations in rhizosphere chemistry under high CO_2_ stress.

**Figure 7:**
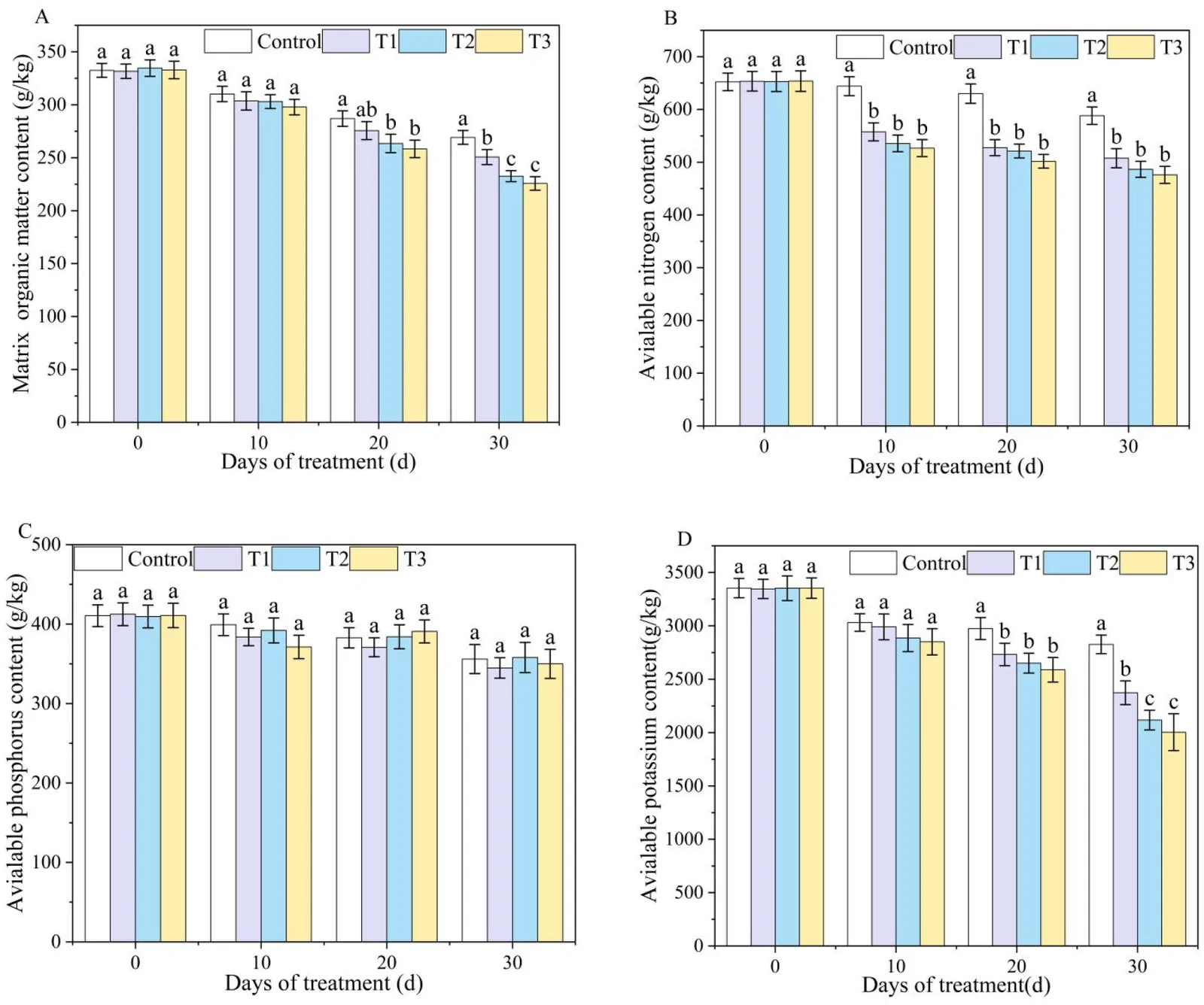
Effect of high soil environmental concentration of CO_2_ on matrix nutrient content in the root zone of oriental melon seedlings. **(A)** Soil organic matter (SOM, g·kg^-^¹); **(B)** Available nitrogen (mg·kg^-^¹); **(C)** Available phosphorus (mg·kg^-^¹); **(D)** Available potassium (mg·kg^-^¹). Sampling times as indicated. Treatments: as in **Figure 2**. Data presentation: Bars = mean ± SD (n = 3). Different letters indicate P<0.05 (ANOVA + Duncan). SOM, soil organic matter.

Changes in rhizosphere soil organic matter (SOM) and available nutrient content in response to elevated root-zone CO_2_ are shown in **Figure 7**. The SOM content (**Figure 7A**) significantly decreased under T2 and T3 treatments by 8.17% and 9.97%, respectively, at 20 days compared to the Control. By day 30, a notable decline was observed across all treatments. Available nutrients exhibited divergent patterns: alkali-hydrolyzable nitrogen (available N, **Figure 7B**) was significantly reduced as early as day 10, although no significant differences were found among the T1, T2, and T3 treatments. In contrast, available phosphorus (available P, **Figure 7C**) remained largely stable throughout the experimental period, indicating its relative insensitivity to the microbial perturbations induced by high CO_2_. Available potassium (available K, **Figure 7D**) began to decline significantly from day 20, with the reduction becoming increasingly pronounced over time. These results demonstrate that elevated root-zone CO_2_ exerts complex and temporally distinct effects on the availability of substrate nutrients.

### 3.5 Elevated root-zone CO_2_ significantly reduced the microbial abundance in the oriental melon rhizosphere

**Figure 8:**
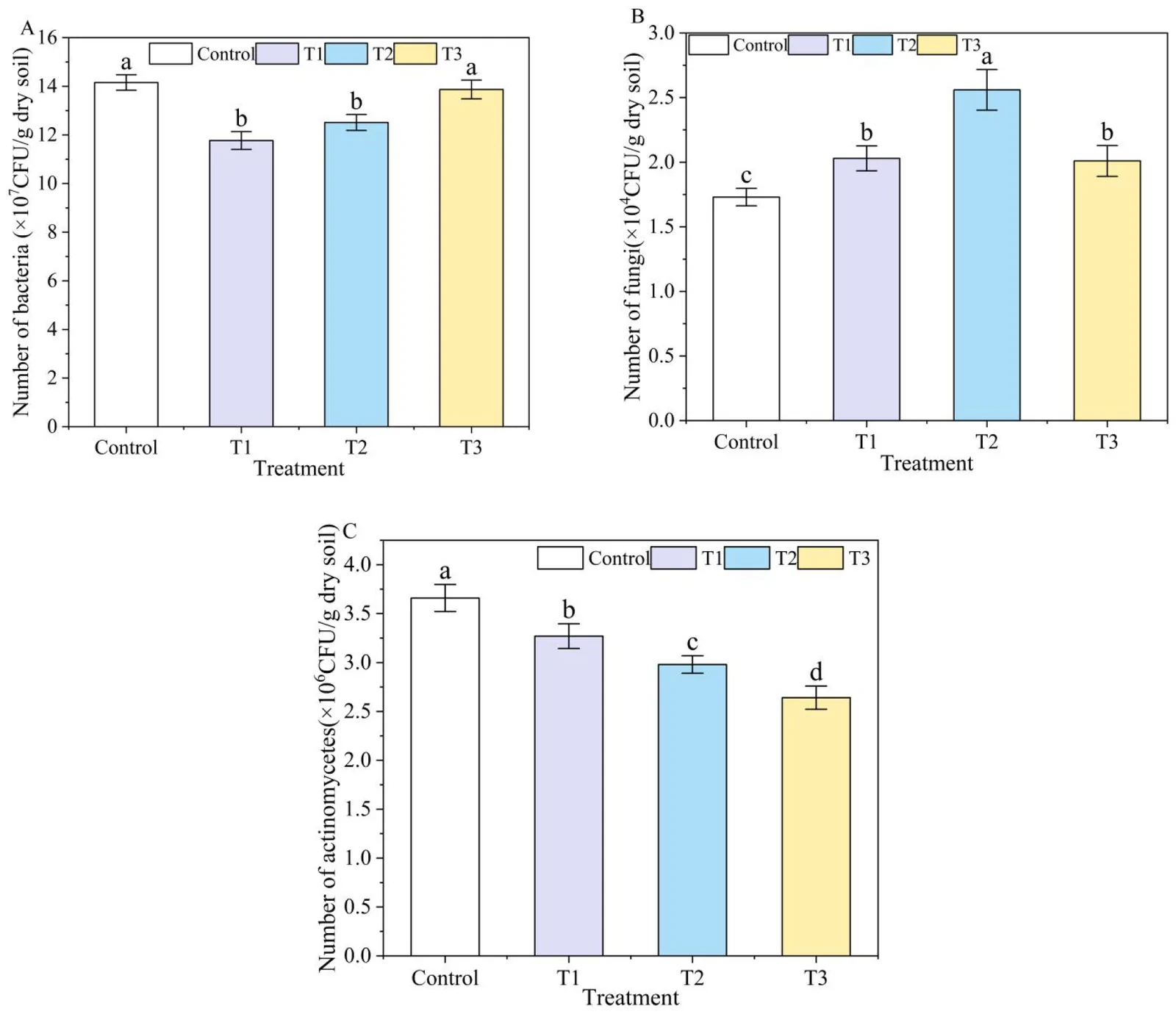
Changes in the number of rhizosphere microorganisms under elevated root-zone CO_2_ concentration. **(A)** Bacteria (×10^7^ CFU·g^-^¹ dry soil); **(B)** Fungi (×10^4^ CFU·g^-^¹); **(C)** Actinomycetes (×10^6^ CFU·g^-^¹). Treatments: as in **Figure 2**. Data presentation: Bars = mean ± SD (n = 3). Different letters indicate significant differences at P<0.05 (ANOVA + Duncan). CFU, colony-forming unit.

The dynamics of rhizosphere microbial abundance under varying elevated root-zone CO_2_ concentrations are shown in **Figure 8**. This study quantified the population sizes of bacteria, fungi, and actinomycetes. The results indicated that bacterial abundance initially experienced suppression followed by a partial recovery as CO_2_ levels increased. The Control group exhibited the highest bacterial count, which was significantly diminished in the T1 treatment. Although a rebound was noted in the T3 treatment, it remained lower than that of the Control group. In contrast, fungal abundance displayed a unimodal pattern, peaking in the T2 treatment, where it was 32.4% and 21.5% higher than in the Control and T3 treatments, respectively. Notably, actinomycete abundance progressively decreased with rising CO_2_ concentrations, showing a 27.9% reduction in the T3 treatment compared to the Control. This trend suggests a lower adaptability of actinomycetes to elevated CO_2_, potentially associated with soil acidification.

### 3.6 Elevated root-zone CO_2_ significantly reduced the alpha diversity of rhizosphere bacteria

**Figure 9:**
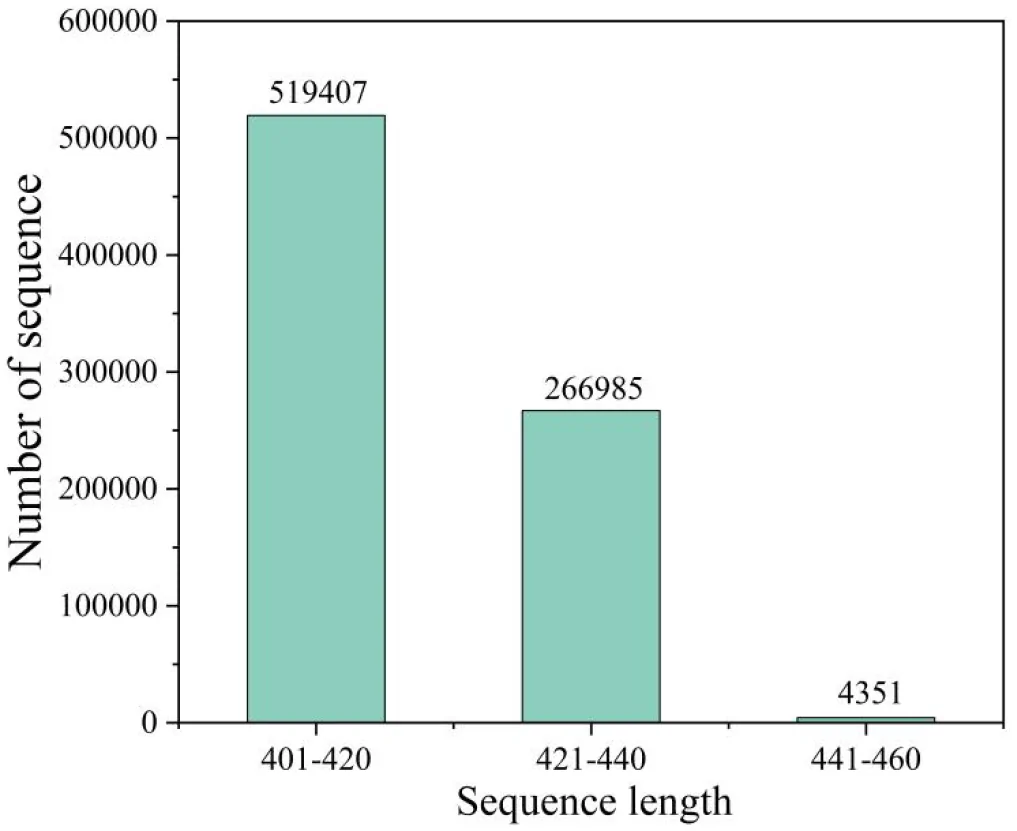
Length distribution of high-quality 16S rDNA sequences from rhizosphere soil samples. The histogram shows the frequency of sequence lengths (bp) across all samples (n = 12). Most sequences (99.93%) are within 401–460 bp. Treatments: Control, T1, T2, T3 (each with three replicates). bp, base pairs.

High-throughput sequencing of the 16S rDNA hypervariable regions generated 791,332 high-quality sequences after stringent quality filtering (**Figure 9**; **Table 4**). Key sequencing metrics indicated that Per-sample reads ranged from 59,030 to 69,672, with a mean of 65,944. The sequences varied in length from 232 to 517 bp, averaging 413.58 bp, and 99.93% of the sequences were concentrated within the 401–460 bp range. These sequencing results demonstrate that the current sequencing depth adequately captures both the taxonomic and structural diversity of the bacterial community present in the root-zone substrate.

**Table 4:** Root zone bacteria 16S rDNA sequencing information table.

Treatment and sample	Seq (reads)	Base (bp)	Mean-length (bp)	Min-length (bp)	Max-length (bp)
Control-1	66663	27511356	412.6930381	266	467
Control-2	65788	27135581	412.4700705	286	453
Control-3	68196	28159856	412.9253329	298	514
T1-1	59030	24380373	413.0166526	265	504
T1-2	60596	25039101	413.213760	327	503
T1-3	66383	27365029	412.2294714	244	492
T2-1	64041	26623564	415.7268625	245	489
T2-2	71109	29341674	412.6295406	305	482
T2-3	67315	27895094	414.396405	283	496
T3-1	62895	26252325	417.3992368	275	517
T3-2	69644	28729683	412.5220119	232	517
T3-3	69672	28822666	413.6908084	247	473

Analysis of 16S rDNA sequencing data demonstrated that elevated root-zone CO_2_ consistently impaired the alpha diversity of the rhizosphere bacterial community (**Figure 9**). Compared to the Control, the elevated CO_2_ treatments (T1, T2, T3) significantly reduced both the Chao1 (richness) and Shannon (diversity) indices of the bacterial community. This reduction indicates that higher CO_2_ concentrations markedly diminished rhizobacterial species richness and community diversity. Although the Shannon index did not show significant differences among the CO_2_ treatments, its declining trend reflected a persistent negative impact. Adequate sequencing depth was confirmed by the non-significant differences in Coverage values across all groups.

The effects of different root-zone CO_2_ concentrations on the β-diversity of the oriental melon rhizosphere bacterial community are presented in **Figure 9**. Principal coordinate 1 (PC1) and principal coordinate 2 (PC2) explained 50.51% and 20.65% of the total community structure variation, respectively, with a cumulative explanation rate of 71.16%. PERMANOVA analysis revealed significant differences in microbial community structure among treatments (R = 0.306, P = 0.033 < 0.05), indicating that different root-zone CO_2_ concentrations markedly altered the species composition and structure of the rhizosphere microbial community.

**Figure 10:**
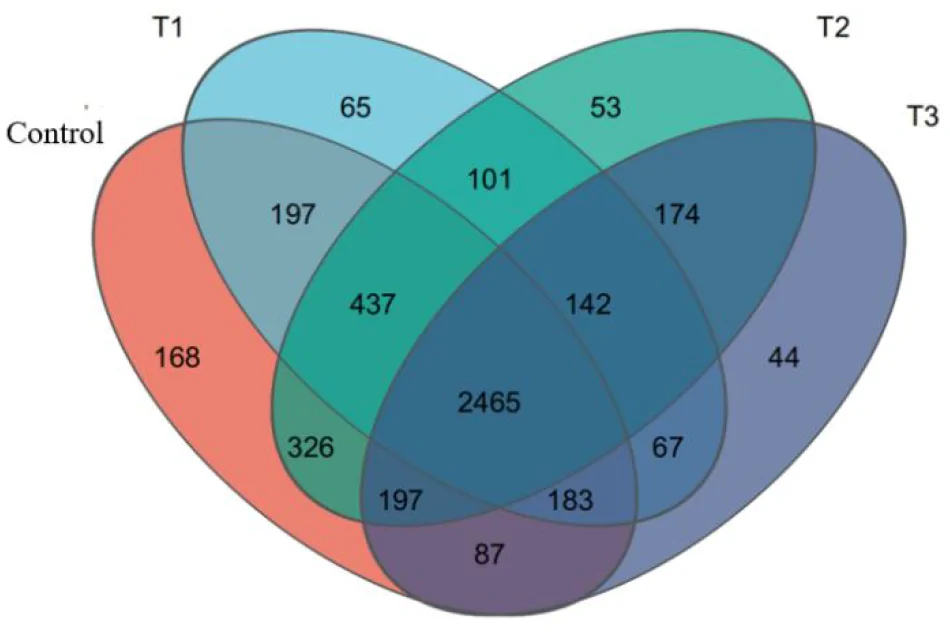
Effective OTU Venn diagram of rhizosphere bacteria. Total OTUs: Control (14,971), T1 (3,425), T2 (3,282), T3 (2,932). Core OTUs shared by all treatments: 2,465. Treatments: as in **Figure 2**. OTU, operational taxonomic unit.

Venn analysis of OTU distribution (**Figure 10**) demonstrated significant alterations in bacterial community composition within the oriental melon rhizosphere under elevated CO_2_ conditions. The total number of OTUs decreased from 14,971 in the Control group to 3,425 in T1, 3,282 in T2, and 2,932 in T3. Only 2,465 OTUs comprised the shared core microbiota across all treatments, while 83.5% (12,506 OTUs) were present in two or more groups. Additionally, the number of OTUs shared with the Control group progressively declined as CO_2_ concentration increased, indicating that the elevated CO_2_ environment diminished the extent of the shared core microbiota within the rhizosphere bacterial community.

In summary, elevated CO_2_ treatment in the rhizosphere significantly reduced both the diversity (Shannon index) and richness (Chao1 index) of the bacterial community associated with oriental melon, while community coverage remained unchanged. This finding further indicates that hypercapnia (i.e., abnormally elevated CO_2_ concentration in the root zone) led to the extinction of certain indigenous bacterial taxa and resulted in a contraction of the shared core microbiota.

### 3.7 Elevated root-zone CO_2_ altered the composition of rhizosphere bacterial communities at the phylum level

**Figure 11:**
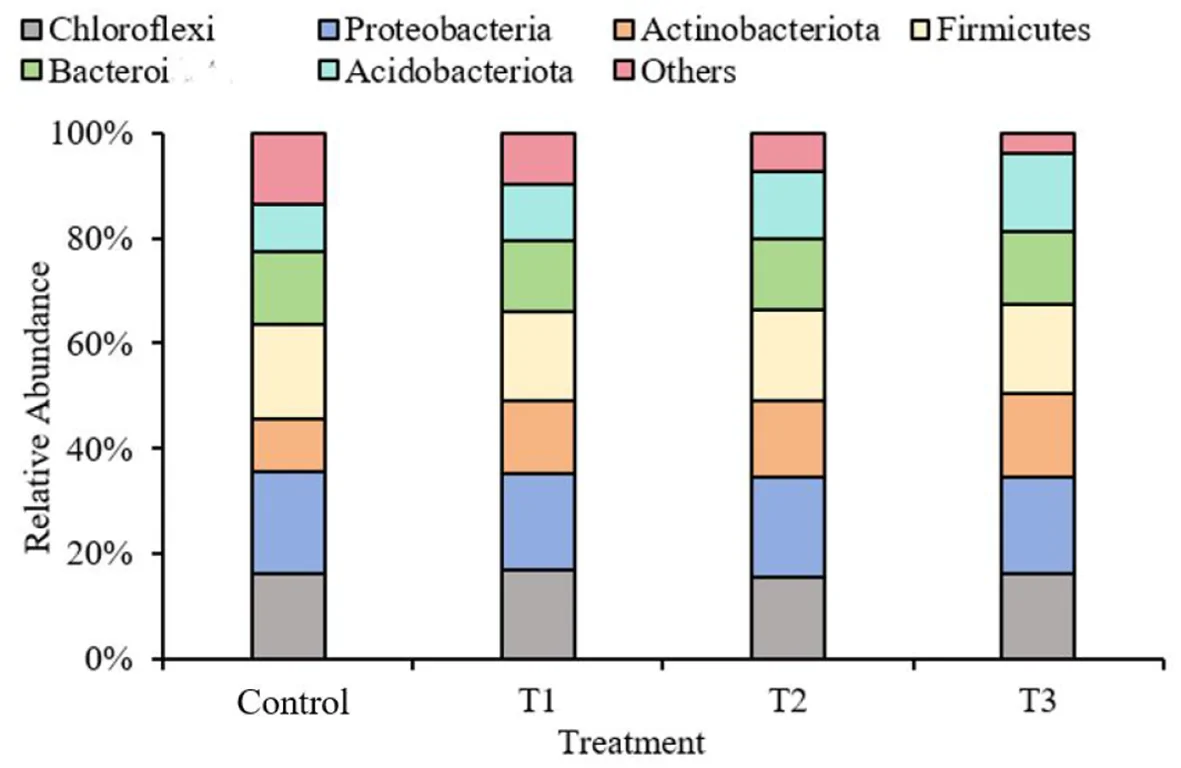
Effective histogram of bacterial community abundance in the rhizosphere at the genus level. Phyla shown: Chloroflexi, Proteobacteria, Actinobacteria, Firmicutes, Bacteroidota, Acidobacteria. Treatments: as in **Figure 2**.

Elevated CO_2_ significantly altered the rhizobacterial community in oriental melon substrate at the phylum level (**Figure 11**). The dominant phyla included Chloroflexi, Proteobacteria, Actinobacteria, Firmicutes, Bacteroidota, and Acidobacteria. While Chloroflexi, Firmicutes, Bacteroidota, and Proteobacteria exhibited no significant changes in relative abundance, both Acidobacteria and Actinobacteria increased in a dose-dependent manner under hypercapnia. This reorganization was mechanistically associated with rhizosphere acidification under elevated CO_2_, which selectively favored acid-tolerant genera such as Acidobacteria and Actinobacteria. These taxa exhibit genomic adaptations that confer resilience to low pH, thereby confirming a community-level eco-physiological response to acidification stress. Nevertheless, the observed community dynamics likely involve more complex feedback loops in the rhizosphere. While elevated CO_2_ drives soil acidification, which may favor acid-tolerant microorganisms, the microbial community itself can substantially influence this process by consuming organic carbon before it is converted into organic acids, thereby modulating the actual intensity and extent of acidification. Consequently, the net outcome of elevated CO_2_ on rhizosphere pH and microbial composition reflects a dynamic interplay between CO_2_-driven acidification and microbial carbon metabolism.

**Figure 12:**
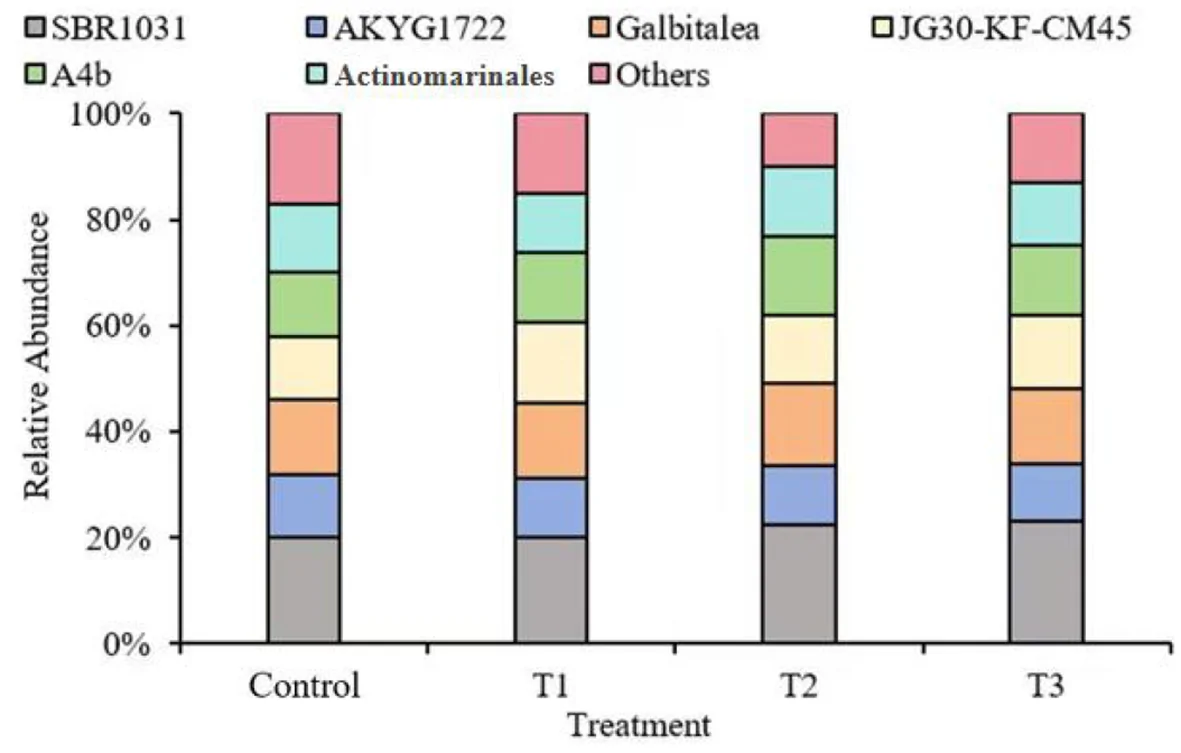
Effective Histogram of bacterial community abundance in the rhizosphere at the genus level. Genera shown: SBR1031, Actinomarinales, A4b, *JG30-KF-CM45*, Galbitalea, AKYG1722. Treatments: as in **Figure 2**.

Taxonomic profiling at the genus level revealed significant alterations in the structure of the rhizobacterial community under elevated CO_2_ (**Figure 12**). Six dominant genera were identified: SBR1031, Actinomarinales, A4b, JG30-KF-CM45, Galbitalea and AKYG1722. Elevated CO_2_ selectively enriched the populations of SBR1031, A4b and Galbitalea. Conversely, Actinomarinales, JG30-KF-CM45 and AKYG1722 maintained stable relative abundances across CO_2_ gradients. This dichotomy highlights niche partitioning between CO_2_-responsive taxa and CO_2_-insensitive taxa.

### 3.8 LEfSe analysis of rhizosphere bacterial communities under elevated root-zone CO_2_ concentration

**Figure 13:**
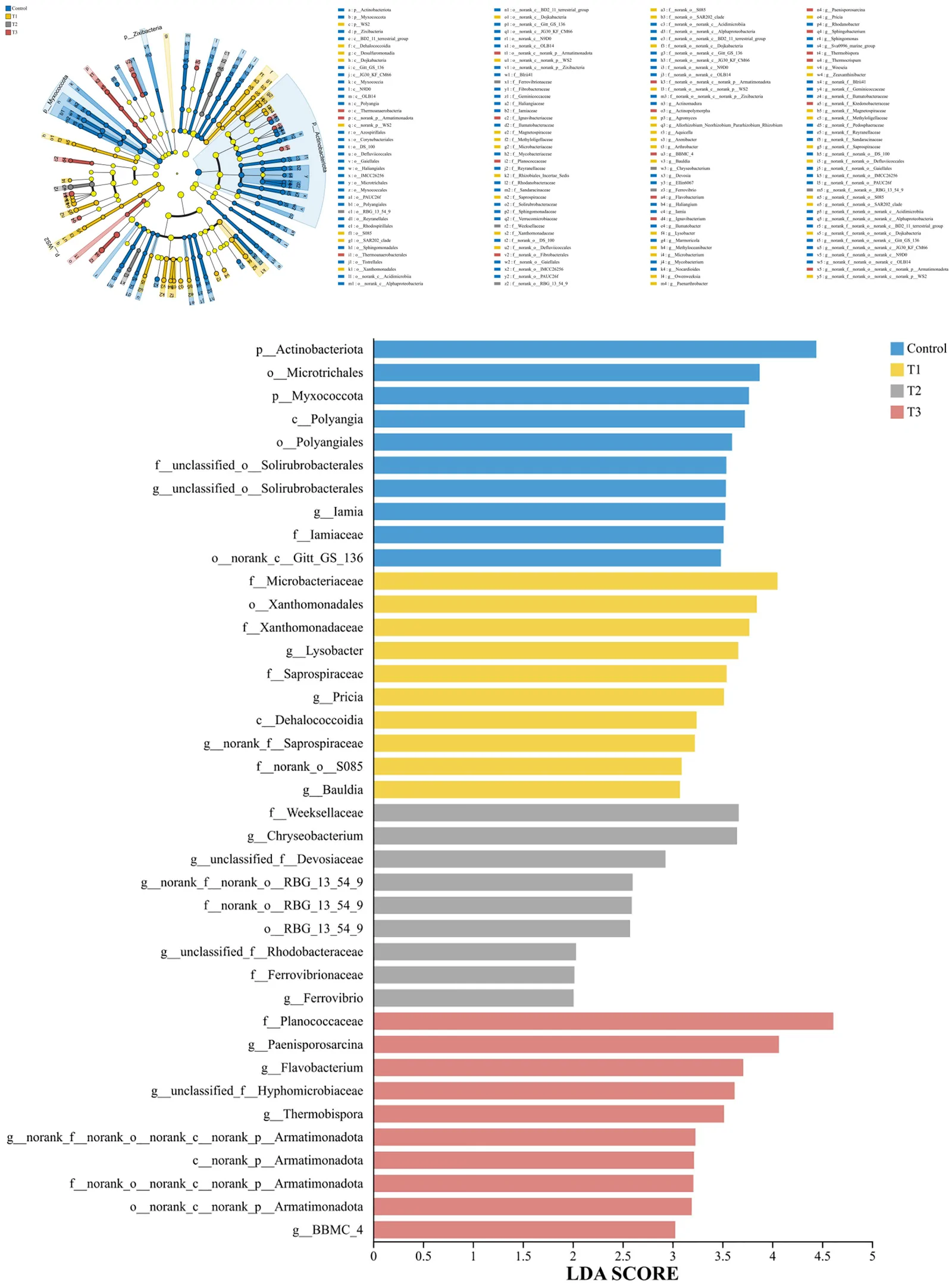
LEfSe analysis of rhizosphere bacterial communities under elevated root-zone CO_2_ concentration. Treatments: as in **Figure 2**.

To further explore the species composition and taxonomic hierarchy with significant differences among treatment groups, LEfSe (Linear Discriminant Analysis Effect Size) analysis was performed to dissect the rhizosphere bacterial community, with an LDA threshold set at > 4.0. As shown in **Figure 13**, multiple core bacterial clades were significantly enriched in the control group. With increasing CO_2_ concentration (T1, T2, T3), distinct biomarkers successively emerged in the community along the gradient. Specifically, T1 enriched several taxa at the family and genus levels. In the T2 and T3 groups, differentially abundant taxa with high LDA scores were conspicuously enriched. Notably, multiple taxonomic units in the T3 treatment exhibited extremely high LDA scores, indicating that high CO_2_ concentration exerted a strong enrichment effect on these specific bacterial taxa. 

### 3.9 Correlation analysis between root-zone bacterial abundance and rhizosphere substrate physicochemical properties

Correlation analysis revealed the relationship between bacterial community composition and soil properties under elevated CO_2_ conditions (**Tables 5**, **6**). At the genus level, Actinomarinales showed a significant positive correlation with pH (r = 0.84, P < 0.05), while A4b exhibited a significant negative correlation with available potassium (r = −0.81, P < 0.05). Galbitalea was positively correlated with available potassium (r = 0.735, P < 0.05), electrical conductivity (r = 0.748, P < 0.05), and pH (r = 0.800, P < 0.05). In contrast, AKYG1722 showed strong negative correlations with available potassium (r = −0.857, P < 0.05), electrical conductivity (r = −0.82, P < 0.05), pH (r = −0.85, P < 0.05), and soil organic matter (r = −0.88, P < 0.05).

**Table 5:** Correlation between rhizosphere bacteria and substrate physical and chemical properties under high concentration of CO_2_ in rhizosphere (Genus level).

	Alkali-hydrolyzable nitrogen	Available phosphorus	Available potassium	EC	pH	Matrix organic matter
*SBR1031*	0.163	-0.393	0.257	0.25	0.194	0.334
*Actinomari-nales*	0.58	0.413	0.342	0.45	0.84*	0.585
*A4b*	-0.39	-0.324	-0.81*	-0.342	-0.53	-0.36
*JG30-KF-CM45*	0.362	0.355	0.243	0.358	0.435	0.53
*Galbitalea*	0.408	0.11	0.735*	0.748*	0.800*	0.57
*AKYG1722*	-0.56	-0.46	-0.857*	-0.82*	-0.85*	-0.88*

Pearson correlation coefficients (two-tailed test) are shown. n=12 (4 treatments × 3 replicates). * P<0.05.

**Table 6:** Correlation between rhizosphere bacteria and substrate physical and chemical propertiesunder high concentration of CO_2_ in rhizosphere (Phylum level).

	Alkali-hydrolyzable Nitrogen	Available Phosphorus	Available Potassium	EC	pH	Matrix organic matter
*Chloroflexi*	0.163	-0.393	0.257	0.25	0.194	0.334
*Acidobacteriota*	0.58	0.413	0.342	0.45	0.84*	0.585
*Proteobacteria*	-0.39	-0.324	-0.81*	-0.342	-0.53	-0.36
*Firmicutes*	0.362	0.355	0.243	0.358	0.435	0.53
*Bacteroidota*	0.408	0.11	0.735	0.748	0.800	0.57
*Actinobacteriota*	-0.56	-0.46	-0.857*	-0.82*	-0.85*	-0.88*

Pearson correlation coefficients (two-tailed test) are shown. n=12 (4 treatments × 3 replicates). * P<0.05.

At the phylum level, Actinobacteriota demonstrated significant negative correlations with available potassium (r = −0.857, P < 0.05), electrical conductivity (r = −0.82, P < 0.05), pH (r = −0.85, P < 0.05), and soil organic matter (r = −0.88, P < 0.05). Proteobacteria showed a significant negative correlation with available potassium (r = −0.81, P < 0.05). These results suggest that elevated root-zone CO_2_ likely influences the abundance of specific microbial taxa by modifying key soil properties, including pH, electrical conductivity, and nutrient availability.

## 4 Discussion

The influence of root-zone CO_2_ concentration on plant nutrient status is intricate and dependent on factors such as plant species, soil characteristics, and mineral nutrient dynamics (Al-traboulsi et al., 2012). Elevated root-zone CO_2_ is known to negatively impact the absorption and translocation of mineral nutrients, resulting in reduced biomass accumulation and lower tissue nutrient concentrations, as demonstrated in various horticultural and agronomic crops (Loladze, 2014). Consistent with studies on carrot (Islam et al., 1998b) and sweet potato (Kitaya et al., 1992; Islam et al., 1997, 1998), our findings in melon demonstrate a significant suppression of growth and nutrient content under elevated root-zone CO_2_, indicating a pervasive carbon overload effect. In contrast, the response varies between legumes and cereals, as illustrated by the inhibited growth in soybean compared to the unaffected rice (Boru et al., 2003). This variation, along with the observed reduction in nutrient content, likely arises from an interplay between biomass-driven nutrient dilution and direct impairment of root metabolic activity (Gojon et al., 2023; Loladze, 2014). The decrease in net photosynthetic rate (Pn) under elevated root-zone CO_2_ treatment was accompanied by significant reductions in stomatal conductance (Gs) and intercellular CO_2_ concentration (Ci) (**Figure 4**). This coordinated decline is consistent with stomatal limitation under conditions where ambient leaf CO_2_ is not elevated. However, non-stomatal factors may also play a role. In the present study, the marked decreases in chlorophyll content (**Figure 3**) and nutrient (especially nitrogen) accumulation (**Figure 5**) suggest that the photosynthetic apparatus itself might be impaired. Furthermore, elevated root-zone CO_2_ stress can reduce root-to-shoot hormone signaling and hydraulic conductance, thereby potentially decreasing mesophyll conductance (gm) and Rubisco activity. Therefore, although stomatal limitation may dominate at the early stage, progressive impairment of leaf biochemical and anatomical characteristics is likely to exacerbate the photosynthetic decline over time. Future studies should directly quantify mesophyll conductance and *in vivo* Rubisco kinetics to fully resolve the relative contributions of stomatal versus non-stomatal limitations under root-zone CO_2_ stress.

A high CO_2_ environment in the root zone influences both crop physiological processes and related soil properties, including the structure of its microbial community (Han et al., 2022; Hinsinger et al., 2009; Helliwell, 2017; Greenway et al., 2006). The significant acidification (**Figure 6A**), elevated EC (**Figure 6B**), and reduced nutrient availability (**Figure 7**) observed under high root-zone CO_2_ result from its dissolution in the soil solution. This process generates carbonic acid, which dissociates to release H^+^, thereby directly lowering the pH. The degree of acidification increases with both rising CO_2_ concentration and treatment duration due to the accumulation H^+^ (Gojon et al., 2023). The increase in soil solution electrical conductivity (EC) under elevated CO_2_ levels indicates a higher total ion concentration, which is influenced by acid dissociation and mineral weathering (Loladze, 2014). The buffering capacity of the substrate plays a crucial role in modulating the pH response; specifically, the weak base effect from carbonate dissolution is generally counteracted by the predominant acidification due to H^+^, resulting in a net loss of pH. This phenomenon is particularly pronounced in poorly buffered, acidic substrates where the depression of pH is most significant (Gojon et al., 2023; Zhang et al., 2018; Patil, 2012). The decrease in pH induced by CO_2_ further affected the availability and speciation of soil nutrients. In environments with lower pH, the solubility of specific nutrient elements may increase (Loladze, 2014). The responses of pH and EC demonstrated a clear dose-dependent relationship, intensifying with rising CO_2_ concentrations until reaching a potential saturation point, likely due to the depletion of soil buffering systems or the establishment of a new chemical equilibrium.

High root-zone CO_2_ lOevels selectively regulated the soil microbiome. Bacterial populations underwent transient suppression, reaching a minimum at T1 before recovering, while fungal populations peaked and subsequently declined, remaining consistently elevated compared to the Control. In contrast, actinomycetes exhibited a steady decline as CO_2_ concentration increased (**Figure 8**). Some studies indicate that elevated CO_2_ levels decrease soil pH and inhibit microbial activity (West et al., 2011; Morozova et al., 2011), while others report an increase in microbial abundance under similar conditions (Wang et al., 2021). This discrepancy can be attributed to the temporal dynamics of stress: a short-term increase in CO2 may stimulate heterotrophic organisms through carbon enrichment, whereas prolonged exposure is likely to promote shifts in community structure due to the significant selection pressures imposed by acidification and other related environmental changes.

**Figure 14:**
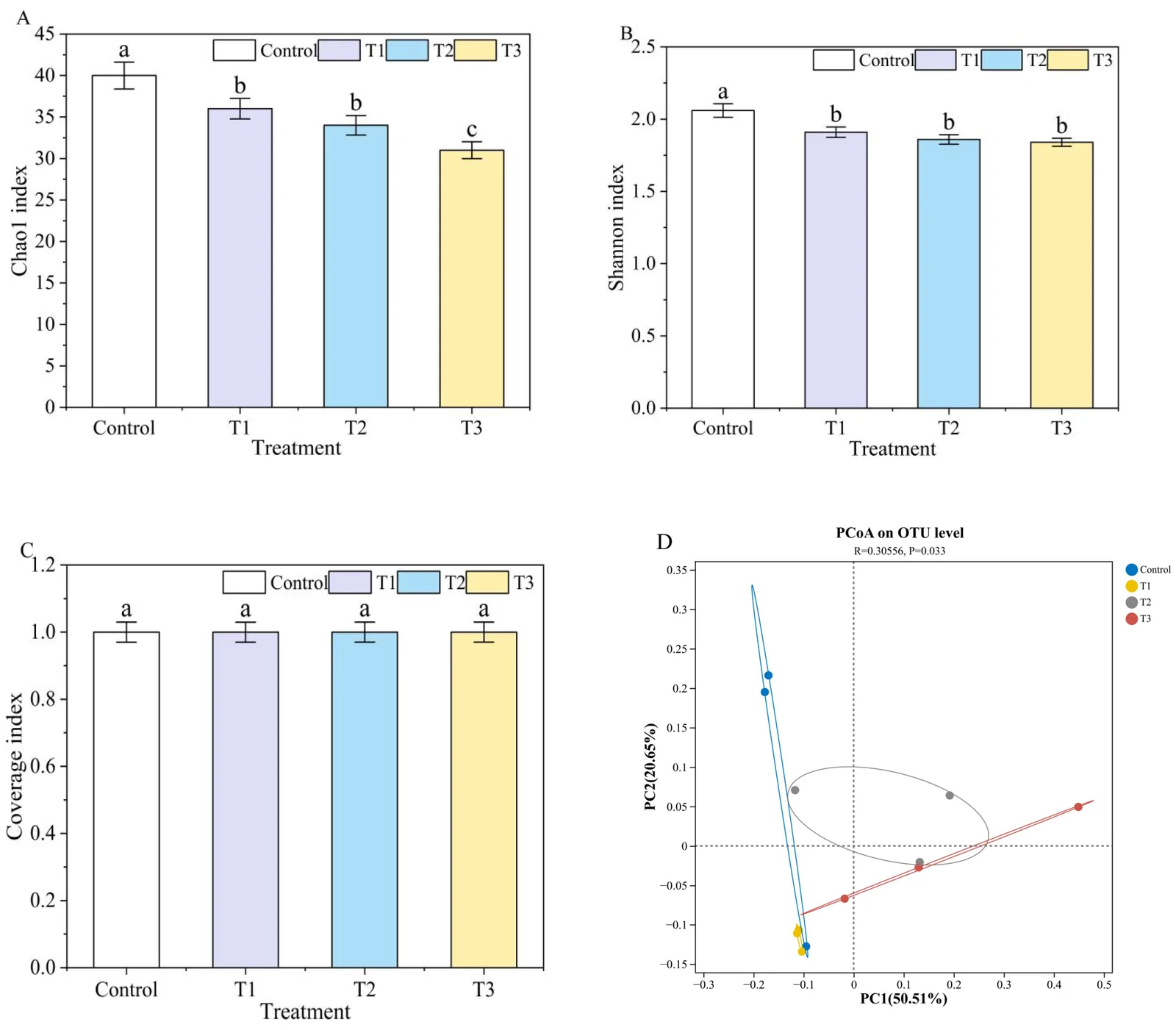
Alpha diversity indices of rhizosphere bacterial communities under different root-zone CO_2_ concentrations. **(A)** Chao1 richness index; **(B)** Shannon diversity index; **(C)** Coverage index. **(D)** β-diversity, In the figure, each point represents an individual biological replicate (n = 3), with different colors indicating different treatments. The ellipses represent 95% confidence intervals for each treatment group. The PERMANOVA (Adonis) test results are: R = 0.3056, P = 0.033. PC1 and PC2 explained 50.51% and 20.65% of the total variation, respectively. Treatments: as in **Figure 2**. Data presentation: Box plots or bars represent mean ± SD (n = 3). Different lowercase letters indicate significant differences at P<0.05 (ANOVA + Duncan). OTU, operational taxonomic unit.

Increasing root-zone CO_2_ concentrations progressively diminished the bacterial Chao1 and Shannon indices, while the Coverage index remained constant (**Figure 14**). pH influences root-zone microbial communities by regulating the bioavailability of nutrients and minerals, as well as the rates of litter decomposition (Han et al., 2020; Wang et al., 2024). Chronic exposure to elevated CO_2_ levels leads to soil acidification, which results in the mortality of acid-sensitive microbes and promotes the dominance of acid-tolerant and anaerobic taxa. This shift ultimately reduces species richness and diversity (Sáenz de Miera et al., 2014). Elevated CO_2_ levels reduce substrate pH, creating conditions conducive to acidophiles while inhibiting neutral and alkaliphilic groups (Meng et al., 2019). This modified environment restricts the majority of microorganisms, allowing only a few dominant taxa to thrive. As a result, both community evenness and diversity experience a significant decline. Our sequencing results illustrate the diversity of rhizosphere bacteria through a complex community structure and a relatively even species distribution, which is corroborated by sufficient sequencing depth that captures most OTUs (Zhou et al., 2012). Elevated CO_2_ primarily resulted in a significant reduction or disappearance of indigenous species. At the phylum level, the relative abundance of Acidobacteria and Actinobacteria progressively increased with rising CO_2_ concentrations. At the genus level, SBR1031, A4b, and Galbitalea exhibited increasing relative abundances under elevated CO_2_. Bacterial abundance-physicochemical correlation analysis indicated that these shifts are associated with changes in substrate pH and EC. Collectively, elevated CO_2_ concentrations in the root zone alter substrate properties, leading to microbial community restructuring that ultimately drives a reduction in system-level biodiversity. As a C3 plant, oriental melon relies on the Calvin cycle for carbon fixation, where the initial carboxylation step is catalyzed by Rubisco. Under prolonged exposure to elevated CO_2_, the maximum carboxylation rate and Rubisco content also decrease (Ancín et al., 2024; Ainsworth and Long, 2005).

Our findings highlight the importance of monitoring and managing root-zone CO_2_ accumulation in protected cultivation. To mitigate the negative effects of high root-zone CO_2_, the following strategies could be implemented: (i) Rhizosphere aeration – periodic mechanical loosening of substrate or installation of passive aeration tubes to enhance gas exchange; (ii) CO_2_ monitoring – deployment of low-cost CO_2_ sensors in the root zone to provide early warning of excessive accumulation. Recent studies emphasize that rhizosphere microbiome dynamics play a critical role in mitigating environmental contaminants through bioremediation (Mahendra, 2024), and plant-microbe interactions are increasingly recognized as a promising pathway for alleviating various stress conditions (Aryal, 2024). Integrating such phytoremediation strategies with targeted rhizosphere aeration may offer a synergistic approach to counteract elevated CO_2_ stress in greenhouse production.

Several limitations of this study should be acknowledged. First, the experiment was conducted under pot conditions using a controlled substrate, which may not fully reflect the complexity of open-field production. Second, only one oriental melon cultivar (‘Yu Mei Ren’) was tested; therefore, the genotypic variation in responses to elevated root-zone CO_2_ among different cultivars remains unknown. Third, the treatment duration was limited to 30 days, precluding assessment of the long-term effects on plant growth, rhizosphere succession, and CO_2_ acclimation. Fourth, although the greenhouse environment is typical for protected cultivation in northern China, natural fluctuations in light, temperature, and humidity may influence the magnitude of the observed effects. Future studies should extend the treatment period, include a wider range of cultivars, and validate the findings under field conditions. In this study, we analyzed the microbial community using OTU-based approaches have inherent limitations. The 97% similarity threshold for OTU clustering tends to collapse multiple distinct organisms into a single unit regardless of their functional or ecological (dis)similarity, thereby underestimating true diversity (Callahan et al., 2017). Nevertheless, there are also relevant evaluation conducted in 2023 on a large-scale clinical cohort, which systematically compared OTU clustering, DADA2, and Deblur, demonstrated that although the three approaches yielded considerable differences in the number of features and alpha-diversity indices, they produced broadly convergent taxonomic profiles and consistent biological conclusions (Liu et al., 2023).

## 5 Conclusions

We conclude that elevated root-zone CO_2_ suppresses melon nutrient uptake via two complementary mechanisms: direct toxicity to root metabolism and indirect effects through rhizosphere microbiome shifts. Elevated CO_2_ inhibited growth and nutrient uptake as early as day 10, preceding progressive soil community succession, which featured decreased overall diversity and taxon-specific changes—bacterial minima at T1, fungal peaks at T2, and dose-dependent actinomycete decline. This restructuring consistently favored acid-tolerant taxa (Acidobacteria, Actinobacteria, and SBR1031). Strong correlations between plant nutrient acquisition and microbial shifts underscore an integrated plant–microbe response to CO_2_ stress, providing insights for adaptation mechanisms and rhizosphere management.

## Data Availability

The datasets presented in this study can be found in online repositories. The names of the repository/repositories and accession number(s) can be found below: NCBI Sequence Read Archive (SRA), BioProject accession number PRJNA1478070.
